# Inbreeding without consequences? Limited effects of inbreeding on life-history and performance traits in *Hermetia illucens*

**DOI:** 10.1186/s12862-026-02609-6

**Published:** 2026-11-03

**Authors:** Patrick Klüber, Rocio Rodriguez-Servin

**Affiliations:** Department of Bioresources, Fraunhofer Institute for Molecular Biology and Applied Ecology, Giessen, Germany

**Keywords:** Black soldier fly, Colony maintenance, Fecundity, Insect farming, Mass rearing, Inbreeding depression, Domestication, Reproductive fitness, Life-history traits

## Abstract

**Background:**

The black soldier fly (*Hermetia illucens*) is key to the circular bioeconomy, but intensive mass-rearing risks genetic drift and inbreeding depression. While early domestication causes rapid genetic erosion, long-term colonies often persist without collapsing, potentially due to the purging of deleterious alleles. Whether highly domesticated strains retain susceptibility to residual inbreeding depression remains unclear. This study evaluated how acute full-sib mating impacts life-history, physiological, and reproductive traits in a laboratory strain colonized for over 64 generations.

**Results:**

Full-sib mating triggered no severe phenotypic declines or compromise growth curves. Developmental durations differed significantly across most ontogenetic stages, but absolute variations were marginal and remained within 1.2 days. Inbred larvae exhibited higher substrate reduction and lower feed conversion efficiency (ECI), indicating elevated metabolic costs. Final larval and adult weights were reduced in the inbred group, although natural sexual dimorphism remained the primary driver of body weight variance. Reproductive parameters and fecundity did not exhibit significant differences between groups. Clutch sizes and oviposition patterns only differed descriptively. In contrast, adult survival was severely impacted; inbred flies showed reduced lifespans and an earlier median lethal time (LT_50_) in females.

**Conclusions:**

Acute inbreeding does not cause an immediate production collapse in long-term domesticated strains, demonstrating phenotypic resilience that is highly valuable for managing sudden population bottlenecks. Nevertheless, metabolic inefficiencies and reduced adult longevity indicate that residual inbreeding depression persists and can compromise survival and resource utilization. These findings provide a foundational population biology perspective for optimizing the genetic management and long-term stabilization of industrial insect rearing systems.

## Background

The black soldier fly, *Hermetia illucens* (BSF; Diptera: Stratiomyidae), has established itself as an important species in circular bioeconomy due to its ability to convert a wide variety of organic substrates into protein- and fat-rich biomass for diverse applications, without being a vector to pathogens or a nuisance to animals or humans [1]. Over the past decades, research on the biology, physiology, and rearing systems of BSF has increased substantially. Studies have addressed nutritional requirements, substrate optimization, larval performance and the environmental conditions affecting the rearing conditions [2–4]. However, only few studies have assessed the performance and adaptation of wild-type populations to industrial rearing systems [5]. One of the factors that can influence the performance of BSF is genetic makeup, specifically genetic drift, defined as a random change in allele frequency within a population. In small populations, genetic drift can reduce genetic diversity. An increased homozygosity may expose deleterious recessive alleles and reduce heterozygote advantage at loci where overdominance occurs, potentially causing inbreeding depression [6, 7]. In insects, inbreeding depression has been shown to negatively impact life-history and physiological traits, such as developmental periods and fecundity in the house fly *Musca domestica* (Diptera: Muscidae) used for food and feed purposes as well as the Mexican fruit fly *Anastrepha ludens* (Diptera: Tephritidae) reared in mass for purposes of the sterile insect technique [8].

In this sense, Hull et al. [9] demonstrated that during early domestication of BSF populations undergoing selection for larval weight, a rapid genetic diversity loss occurred. A single nucleotide polymorphism (SNP)-based analysis revealed that reduced heterozygosity and less phenotypic variability occur within only three generations. Such findings provide evidence that drift and inbreeding could dominate early selection phases and influence production traits [9]. Similarly, Rhode et al. [10] revealed that newly established BSF colonies stemming from few founders, experience rapid genetic erosion and population decline within only five generations [8, 10]. This emphasizes the possibility that combined effects of inbreeding and genetic drift eventually lead to colony collapse [11].

In contrast to these studies, where the focus lies on wild caught or early domesticated populations, mass-reared BSF lines in use today have been maintained for multiple generations without collapsing [12, 13]. Underlying mechanisms could be either a purge of deleterious alleles or compensation under domesticated conditions [14]. It remains unclear whether a full-sib mating can further increase inbreeding in long-maintained BSF lines and, if so, this is associated with changes in key fitness related and economically important traits.

Evidence for effects of inbreeding depression in BSF remains limited. In previous studies, reduced pupal survival after selective inbreeding has been observed, while larval growth was less affected [15], suggesting that certain traits may differ in their response to inbreeding. This emphasizes the need for a comprehensive and exhaustive evaluation of multiple traits across all life stages to assess fitness consequences or effects of inbreeding [11].

Physiological and temporal life-history traits, such as larval developmental time, survival rate, growth rate and oviposition parameters have been previously evaluated to provide an overview of the production efficiency in BSF [16, 17]. In the context of inbreeding, changes in these traits are commonly interpreted as evidence of inbreeding depression, reflecting the expression of deleterious recessive alleles or loss of heterozygosity [18]. Therefore, quantifying life-history traits offers a robust approach to detect and characterize potential fitness costs associated with reduced genetic diversity [11].

Despite its practical importance, the extent to which long-term colonized BSF populations remain susceptible to inbreeding depression has not been evaluated. It remains unclear whether additional inbreeding in already domesticated lines results in further performance declines across ontogenetic stages. Therefore, this study aimed to determine whether full-sib mating produces measurable fitness costs affecting the life-history, physiological, and reproductive traits of a long-term laboratory-reared BSF population. Utilizing a base population maintained for over 64 generations under stable laboratory scale provided a unique opportunity to evaluate the threshold at which a full-sib mating triggers phenotypic consequences. Specifically, the objectives of this study were:

1) To evaluate the effects of acute full-sib mating on developmental duration and survival throughout the entire BSF life cycle.

2) To quantify the impacts of inbreeding on growth performance, resource utilization, and morphological traits.

3) To assess the consequences of inbreeding on reproductive fitness, focusing on oviposition phenology, fecundity, and adult longevity dynamics.

## Results

### Temporal and life-history traits

**Fig. 1:**
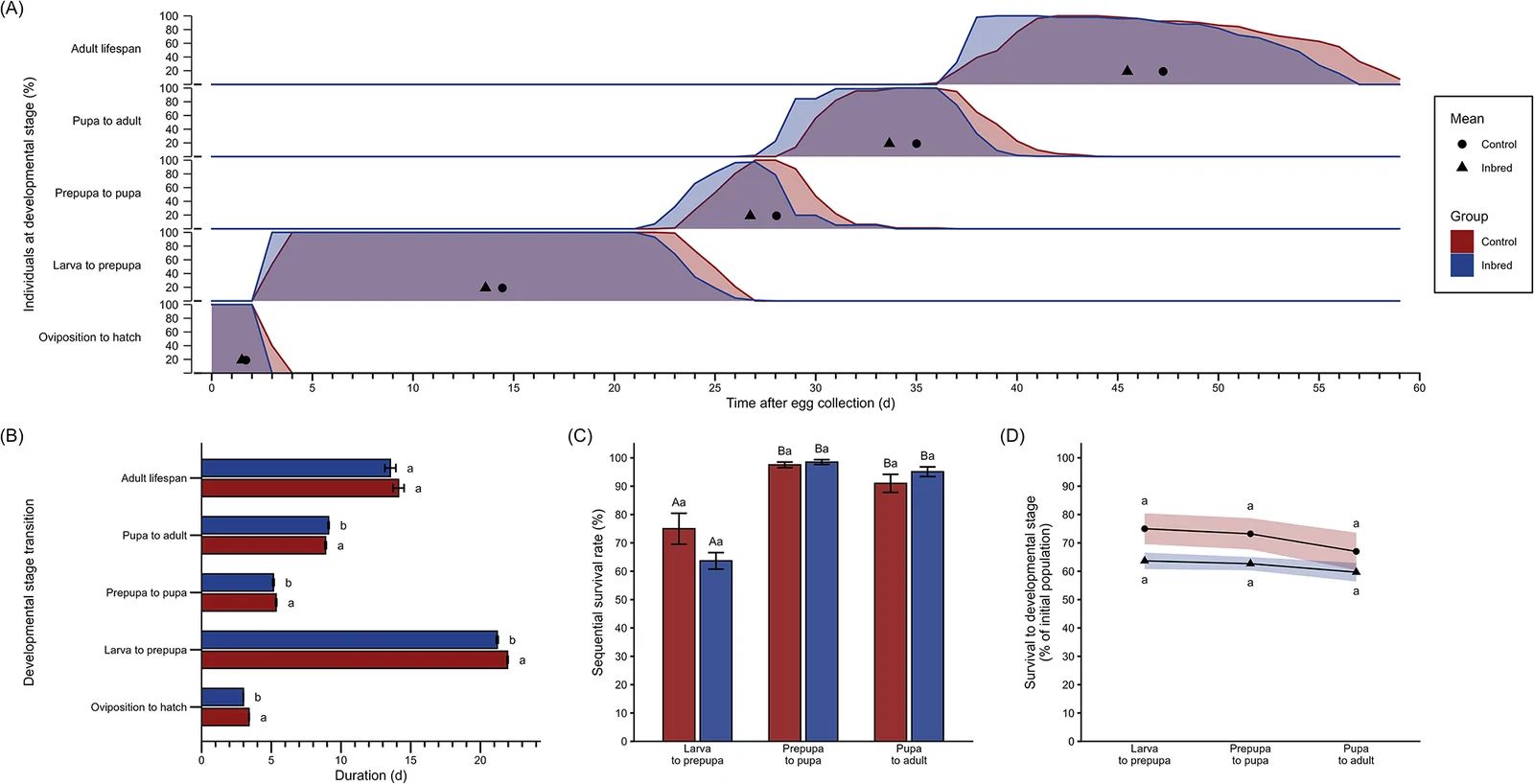
Effects of inbreeding on developmental duration, survival, and ontogenetic stage transitions in *H. illucens*. (**A**) Temporal course of developmental stages based on the percentage of individuals present. (**B**) Mean duration of each developmental stage. (**C**) Sequential survival rate across stage transitions expressed as a percentage of the preceding stage population. (**D**) Overall survival rate expressed as a percentage of the initial neonate population. Data represent mean ± SE of five biological replicates (error bars in panels (**B–C**); shaded area in panel (**D**)). Statistical differences were assessed using Mann–Whitney U tests (**B**), two-way ANOVA followed by Tukey’s post hoc test (**C**), and two-sample t-tests (**D**). Uppercase letters indicate significant differences among developmental stages; lowercase letters indicate significant differences between groups (*p* < 0.05). In (**D**), lines connect the observed stage-specific means for each group and serve as visual guides. (**A–D**) Besides oviposition to hatch (*n* = 10), all data shown were obtained from populations of *n* = 100 individuals

Developmental duration differed significantly between the control and inbred groups across all ontogenetic stages, except for adult lifespan. Although mean differences between the groups never exceeded 1.2 days, control individuals developed more slowly than their inbred counterparts throughout development (Fig. 1A–B). For instance, hatching time exhibited higher variance in the control group (3.4 ± 0.5 d, SD) but remained uniform (3.0 ± 0.0 d, SD) in the inbred group (*n* = 10; Z = 12.64; *p* < 0.0001). Larval development was significantly prolonged in the control group (22.2 ± 0.4 d, SD) compared to the inbred group (21.0 ± 0.0 d, SD; *n* = 5; Z = 6.62; *p* < 0.0001).

During metamorphosis, the prepupal-to-pupal transition spanned 4–6 days (5.0 ± 0.7 d, SD) in the control group, whereas it remained constant (5.0 ± 0.0 d, SD) in the inbred line (*n* = 5; Z = 2.67; *p* = 0.007). A similar pattern emerged during adult eclosion; intra-group variability led to a significant difference (*n* = 5; Z = 5.36; *p* < 0.0001), with the pupal-to-adult phase ranging from 8 to 11 days in controls compared to a stable 9 days in the inbred group (Fig. 1B). Total adult lifespan was slightly reduced in inbred flies (13.8 ± 0.7 d, SD) compared to controls (14.9 ± 0.6 d, SD), though this trend was not statistically significant (*n* = 100; Z = 0.98; *p* = 0.32). Importantly, while total average lifespan appeared uniform, fine-scale pairwise comparisons of sex-specific Kaplan–Meier survival functions showed significant differences (see Sect.  2.5).

Independent of these developmental timelines, survival data identified the larva-to-prepupa transition as a critical phase. Although sequential survival during this transition was 11.3% higher in the control group than in the inbred group, the difference was not statistically significant (*n* = 5; F_1, 18_ = 0.49, *p* = 0.48). However, survival rates differed significantly when comparing the larva-to-prepupa transition against subsequent developmental phases (*n* = 5; F_1, 18_ = 0.49; *p* < 0.0001). For both the prepupa-to-pupa and pupa-to-adult transitions, survival exceeded 90.9% in both groups, showing no significant inter-group variation (Fig. 1C). Similarly, overall survival – measured as the percentage of the initial population reaching adulthood – did not differ significantly between groups nor across developmental stages (*n* = 5; t_5.67_ = 1.83; *p* ≥ 0.11; Fig. 1D).

### Growth performance and resource utilization

**Fig. 2:**
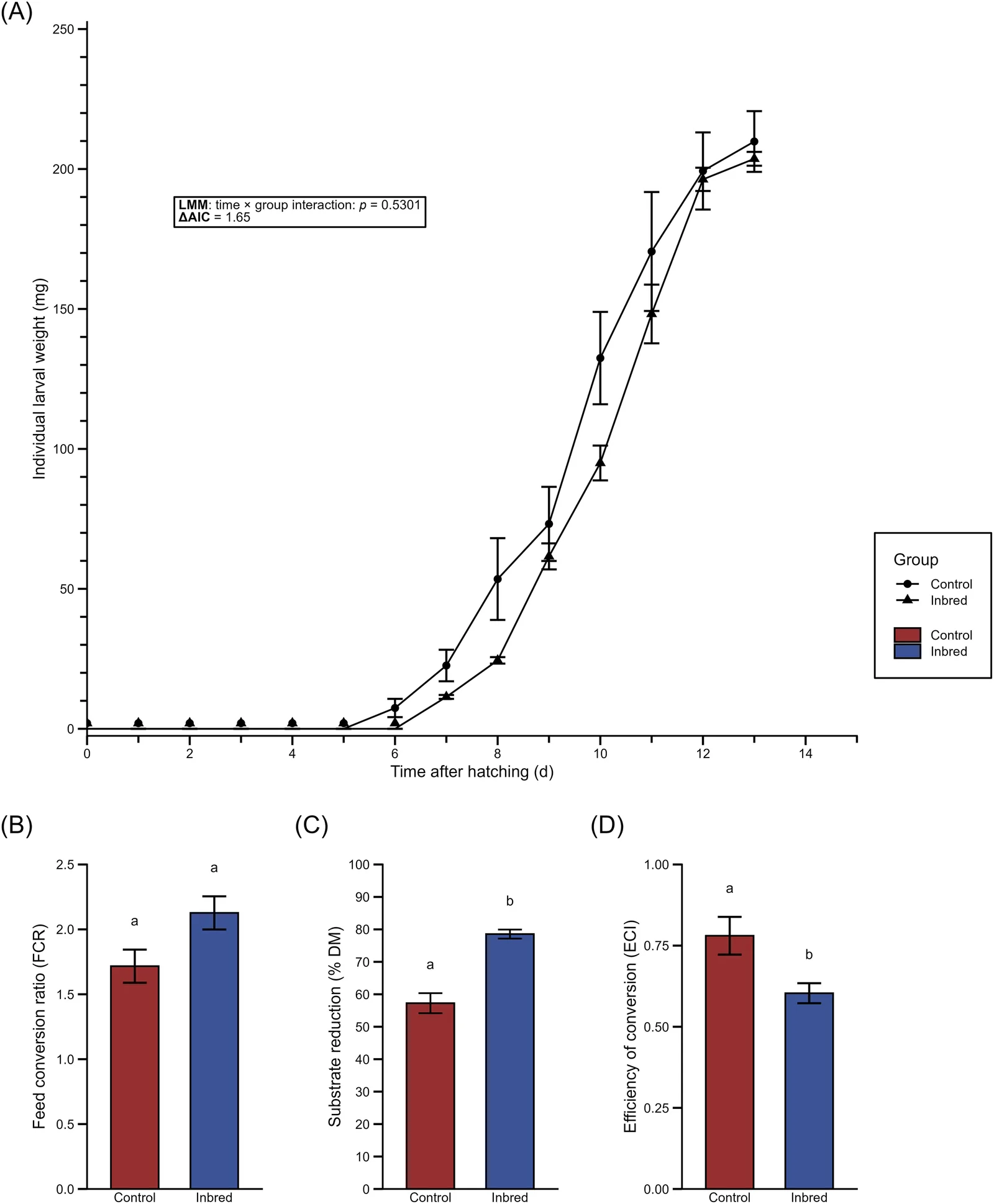
Effects of inbreeding on growth performance and substrate utilization indicators in *H. illucens*. (**A**) Larval growth curves (*n* = 25), (**B**) feed conversion ratio (FCR), (**C**) substrate reduction and (**D**) efficiency of conversion of ingested feed (ECI) for control and inbred groups. Data represent mean ± SE of five biological replicates. Larval growth curves **(A)** were analyzed using a linear mixed model (LMM). Lowercase letters indicate significant differences between groups (*n* = 5; *p* < 0.05), as determined by two-sample t-tests (**B–D**). (DM = dry matter; ∆AIC = Akaike information criterion)

Growth curves did not differ significantly between the groups (*n* = 5; time × group interaction: ΔAIC = − 1.65, *p* = 0.53; Fig. 2A), with both groups exhibiting an identical trend from day 6 through day 12, at which point individual larval weights stagnated at approximately 200 mg. The resource utilization indicators reveal interesting information about the assimilation of the substrate. Although the inbred group exhibited a 23.8% higher feed conversion ratio (FCR) than the control, this difference was not statistically significant (*n* = 5; F_1, 5.34_ = 3.37; *p* = 0.06; Fig. 2B). However, substrate reduction was approximately 37.2% higher in the inbred group than in the control (*n* = 5; F_1, 5.35_ = 10.66; *p* < 0.0001; Fig. 2C). In contrast, the efficiency of the conversion of the ingested feed (ECI) was 29.4% higher in the control group, representing a significant inter-group difference (*n* = 5; F_1, 5.35_ = 4.77; *p* = 0.03; Fig. 2D). Consequently, inbred larvae ingested a higher amount of substrate that was neither assimilated nor incorporated into biomass.

### Physiological indicators across developmental stages

**Fig. 3:**
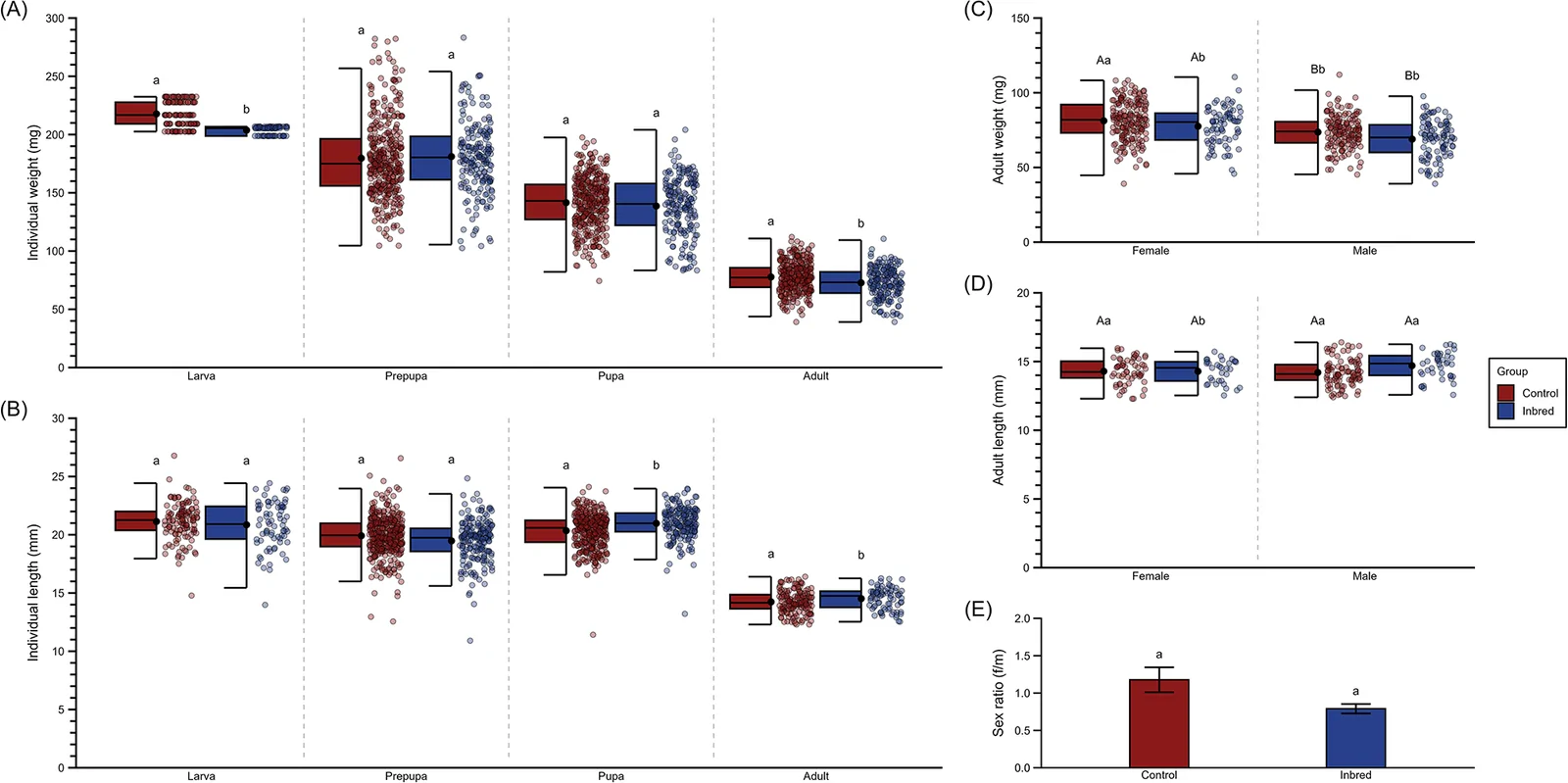
Effects of inbreeding on physiological parameters and sex ratio across *H. illucens* developmental stages. Comparison of individual weight (**A**) and length (**B**) across developmental stages for control and inbred groups (*n* = 100). (**C**) Sex-dependent individual weight and (**D**) length of adult flies (*n* = 50). (**E**) Sex ratio (female/male) for each group (*n* = 5). (**A–D**) Box plots represent the interquartile range (IQR) and median (center lines), means (black dots), whiskers (1.5 × IQR), and individual data points (right dots). Data in panel (**E**) represent mean ± SE of five biological replicates. Statistical analyses were performed using Mann–Whitney U tests (**A**,** B**), two-way ANOVA followed by Tukey’s post hoc test (**C**,** D**) and two-sample t-test (**E**). Uppercase letters indicate significant differences between sexes; lowercase letters indicate significant differences between groups (*p* < 0.05)

With an average of 217.7 mg, control individuals attained a 6.9% higher final larval weight than their inbred counterparts (*n* = 100; Z = 8.68; *p* < 0.0001). However, this discrepancy disappeared during later juvenile stages, showing no significant differences at either the prepupal (*n* = 100; Z = -1.24; *p* = 0.21) or pupal stage (*n* = 100; Z = 0.95; *p* = 0.34), where controls were only 0.2% and 3.2% heavier, respectively (Fig. 3A). For adult weights, a sex-independent comparison revealed a significant inter-group difference (*n* = 100; t_354.85_ = 4.14; *p* < 0.0001), with control flies being 6.6% heavier than inbred individuals (Fig. 3A). Relative to final larval weight, adult weight reflected a proportional weight reduction of ~ 64.5% in both the control and inbred groups.

Developmental lengths were similar between groups during the larval (*n* = 100; Z = 0.75; *p* = 0.44) and prepupal stages (*n* = 100; Z = 2.61; *p* = 0.09) but differed significantly at the pupal stage (*n* = 100; Z = 4.98; *p* < 0.0001). Control larvae and prepupae were 2.4% and 2.0% longer than inbred individuals, respectively, whereas inbred pupae were 3.9% longer than controls. Across juvenile stages, a length reduction of up to 33.2% relative to final larval length was observed in sex-independent adult measurements. Although only 2.8% longer, inbred adults differed significantly from controls (*n* = 100; t_151.21_ = -2.12; *p* = 0.03; Fig. 3B).

Adult physiological indicators varied based on both sex and group. Females were significantly heavier than males across both groups (*n* = 50; F_1, 510_ = 49.54; *p* < 0.0001), with sexual dimorphism in weight ranging from 11.6% in the inbred to 13.2% in the control group. The inter-group comparison confined to females also confirmed significantly higher weights in controls (*n* = 50; F_1, 510_ = 13.19; *p* < 0.0001; Fig. 3C). In contrast to weight, body length did not differ between sexes within the same group (*n* = 50; F_1, 224_ = 1.44; *p* = 0.23). However, adult length differed significantly between control and inbred females (*n* = 50; F_1, 224_ = 3.33; *p* = 0.02), with inbred females being 4.3% longer than control flies (Fig. 3D). No inter-group differences were detected for male weights or lengths. The sex ratio (female/male) ranged from 0.79 to 1.18 and did not differ significantly between groups (*n* = 100; t_124.22_ = 5.01; *p* = 0.08; Fig. 3E).

### Reproductive traits

**Fig. 4:**
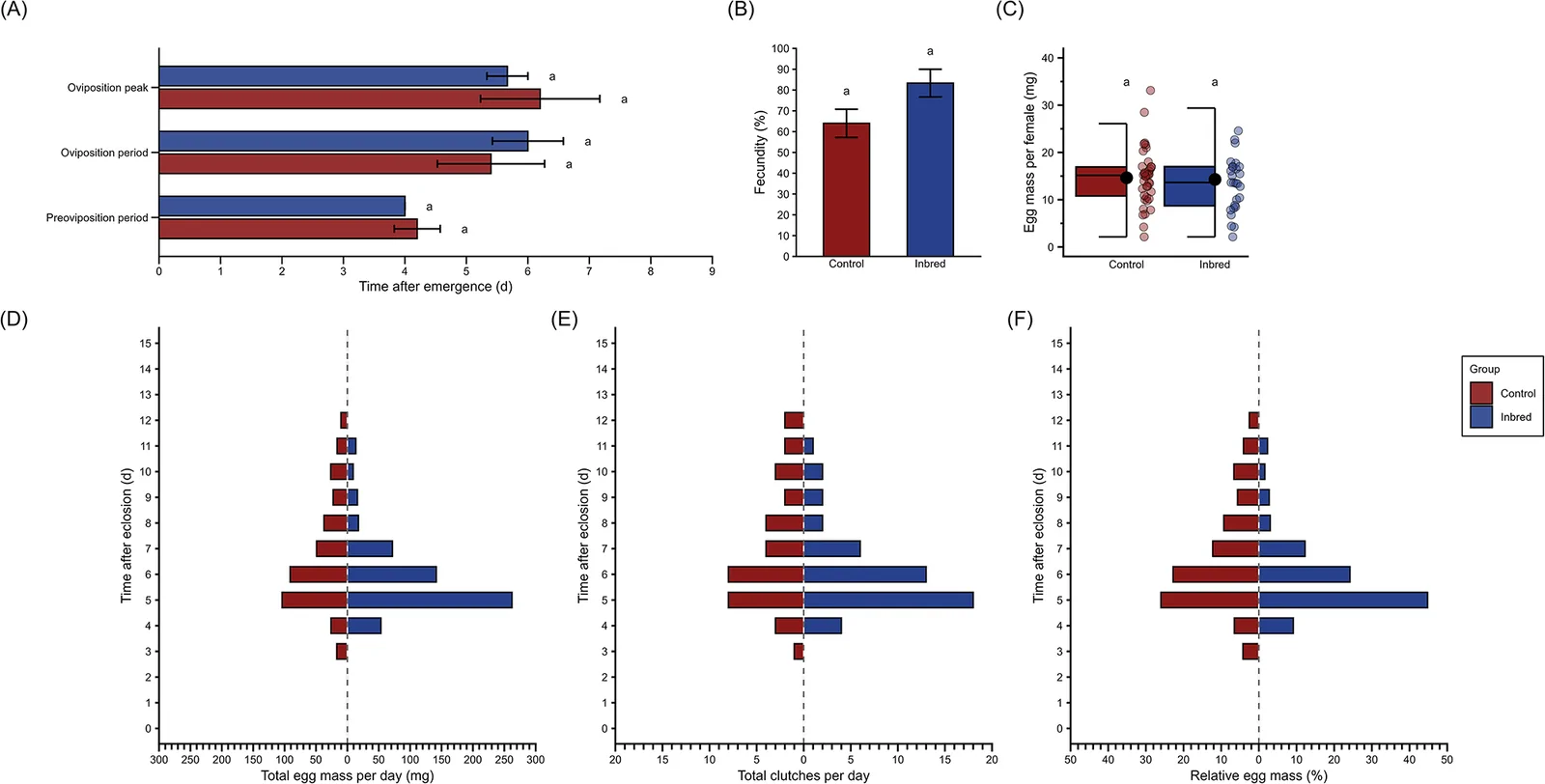
Effects of inbreeding on oviposition timing, fecundity, and egg production in *H. illucens*. (**A**) Temporal oviposition indicators after adult emergence, (**B**) fecundity, and (**C**) total egg mass per female for control and inbred groups. Temporal course of oviposition based on (**D**) the total egg mass per day, (**E**) the total clutches per day, and (**F**) the relative egg mass collected. Data in panels (**A–B**) represent mean ± SE. (**C**) Box plots represent the interquartile range (IQR) and median (center lines), means (black dots), whiskers (1.5 × IQR), and individual data points (right dots). Lowercase letters indicate significant differences between groups (*p* < 0.05), as determined by Mann–Whitney U tests. (**A–F**) All data shown were obtained from female populations of *n* = 50 individuals

The average preoviposition period was 4.2 days for the control and 4.0 days for the inbred group, with the first egg clutches observed at 3- and 4-days post-emergence, respectively. Oviposition peaked at an average of 6.2 days (controls) and 5.7 days (inbred) after emergence, continuing up to day 12 post-emergence for control individuals and day 11 post-emergence for the inbred group (Fig. 4A). Despite these temporal variations, no significant inter-group differences were detected for the preoviposition period (*n* = 5; Z = 0.11; *p* = 0.91), oviposition period (*n* = 5; Z = 0.78; *p* = 0.43), or oviposition peak (*n* = 5; Z = 0.50; *p* = 0.66).

Fecundity, measured as the proportion of females ovipositing, was 29.6% higher in the inbred group than in controls, though this difference was not statistically significant (*n* = 5; Z = 1.53, *p* = 0.12; Fig. 4B). Similarly, mean clutch size (eggs/clutch) and mean clutch weight were 3.4% higher in inbred individuals. Total cumulative clutch weight per female (accounting for multiple ovipositions) did not differ between the groups (*n* = 50; Z = 0.33, *p* = 0.73; Fig. 4C), with inbred females producing 2.5% more egg mass than their control counterparts. Despite these uniform averages, substantial intra-group variability occurred within the recorded ranges. Individual clutch size spanned 45–900 eggs in the inbred group compared to 129–1497 eggs in the control group, corresponding to clutch weights of 1.07–21.60 mg and 0.31–35.91 mg, respectively (*n* = 50).

Throughout the overall 10-day oviposition span for controls (days 3–12 post-emergence) and the 8-day span for the inbred group (days 4–11 post-emergence), the majority of clutches and egg mass were deposited on days 5 and 6 in both groups. Notably, on day 5 post-emergence, the inbred group deposited 2.3-fold more clutches and 2.5-fold higher egg mass than the control group (Fig. 4D–E). Combined oviposition data for days 5 and 6 revealed that control females deposited 48.7% of their relative egg mass during this 48-hour window, whereas inbred females concentrated 69.0% of their relative egg mass within the same two days (Fig. 4F).

**Fig. 5:**
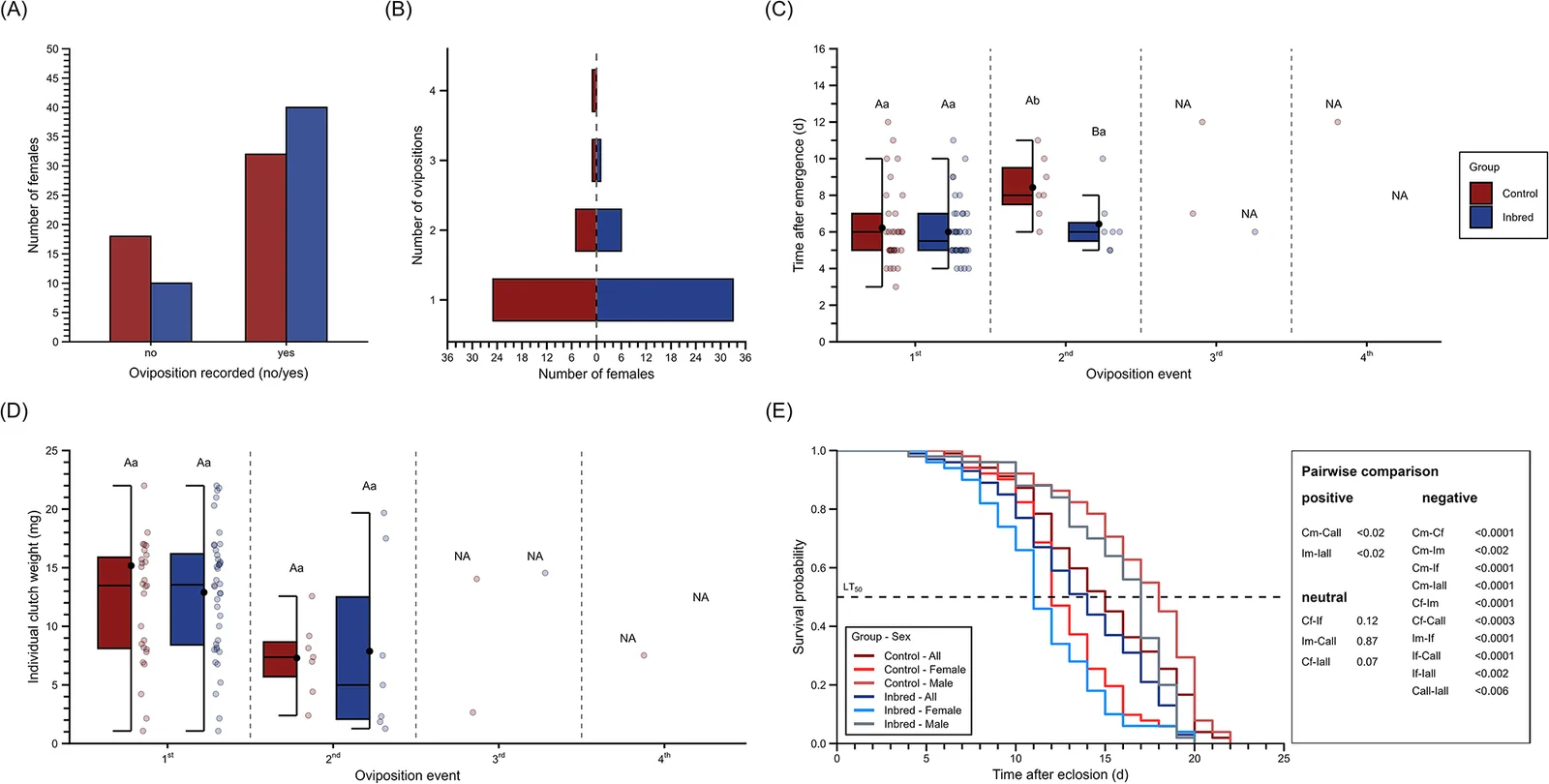
Effects of inbreeding on oviposition dynamics and adult survival in *H. illucens*. (**A**) Number of females with recorded oviposition events (yes/no), (**B**) number of oviposition events per female, (**C**) timing of oviposition events after emergence, and (**D**) egg mass per oviposition event for control and inbred groups. (**E**) Sex-dependent Kaplan–Meier survival functions for both groups (*n* = 100). (**A–B**) Data represent absolute counts (*n* = 50). (**C–D**) Box plots represent the interquartile range (IQR) and median (center lines), means (black dots), whiskers (1.5 × IQR), and individual data points (right dots; *n* = 50). Uppercase letters indicate significant differences among oviposition events; lowercase letters indicate differences between groups (*p* < 0.05), as determined by two-way ANOVA followed by Tukey’s post hoc test **(C–D)**. *p*-values in panel (**E**) indicate pairwise group comparisons via log-rank test, categorized as ‘positive’ (significant increase), ‘negative’ (significant decrease), or ‘neutral’ (no significant difference) following the framework by Klüber et al. [18]. (NA = not applicable; LT_50_ = median lethal time; f = female; m = male; C = control; I = Inbred)

The proportion of females that oviposited at least once was 16% higher in the inbred group than in controls (Figs. 4B and 5A), whereas the total egg mass per female remained similar between the groups (Fig. 4C). Multiple oviposition events occurred in both groups, with at least seven females per group (14% of the respective group totals) ovipositing two or more times. These individuals represented 21.8% and 17.5% of the ovipositing females in the control and inbred groups, respectively (Fig. 5B). Second oviposition events occurred within days 5–11 post-emergence and differed significantly between groups (*n* = 50; F_1, 82_ = 4.14; *p* = 0.04; Fig. 5C). In the control group, the first female with a second oviposition event was detected on day 6 post-emergence, whereas a second oviposition event of an inbred group female was first observed on day 5 post-emergence. Third oviposition events for the control group were detected on days 7 and 12 post-emergence, whereas the only third oviposition event in the inbred group was recorded on day 6. Additionally, a fourth oviposition event was documented for one control female 12 days post-emergence (Fig. 5C).

Clutch weight did not differ significantly between the groups across the first and second oviposition events (*n* = 50; F_1, 67_ = 0.008; *p* = 0.92; Fig. 5D). For the first oviposition, mean clutch weights were 15.17 mg for the control group and 12.90 mg for the inbred group. At the second oviposition, mean clutch weights decreased by 51.9% to 7.29 mg in the control group and by 39.0% to 7.87 mg in the inbred group. For the control group, the third and fourth oviposition events yielded weights ranging between 2.05 and 14.04 mg, whereas the single third oviposition recorded for an inbred female weighed 14.55 mg.

### Survival and longevity

Kaplan–Meier survival analysis confirmed that adult survival did significantly differ between groups (*n* = 100; *χ²* = 71.83; *p* < 0.001; Fig. 5E). A sex-independent comparison between the control and inbred group revealed that the control group lived one day longer than the inbred group (*n* = 100; *χ²* = 7.36; *p* < 0.006). Furthermore, sex-specific survival patterns were found, with males consistently showing up to 30.8% longer lifespans than females in both the control (*n* = 50; *χ*² = 33.25; *p* < 0.0001) and inbred (*n* = 50; *χ*² = 24.66; *p* < 0.0001) groups. Interestingly, inbred females did not differ significantly in longevity from control females (*n* = 50; *χ*² = 2.30; *p* = 0.12), but control males lived 7.1% longer than inbred males (*n* = 50; *χ*² = 9.74; *p* = 0.002).

LT_50_ also differed between groups (*n* = 5; *χ*² = 8.80; *p* = 0.003), with the control group having a 5.8% longer LT_50_ than the inbred group. Regarding sex-specific survival patterns, males showed a > 50% higher LT_50_ value than females in both the control (*n* = 5; *χ*² = 22.27; *p* < 0.0001) and inbred (*n* = 5; *χ²* = 27.14; *p* < 0.0001) groups. In contrast to total longevity, the inbred female LT_50_ was significantly earlier than the control LT_50_ by 8.3% (*n* = 5; *χ*² = 11.87; *p* < 0.0001), whereas the male LT_50_ for the control and inbred groups showed no significant difference at 5.5% (*n* = 5; *χ²* = 2.50; *p* = 0.11).

## Discussion

Previous studies demonstrate that the BSF can suffer from domestication-induced inbreeding depression, resulting in reduced population fitness [5, 9, 10]. Here, we evaluated the impacts of full-sib mating on the life-history, physiological, and reproductive traits of a BSF strain previously domesticated for over 64 generations. Our objective was to determine whether a single generation of full-sib mating triggers further inbreeding depression, or if performance remains stable. Unchanged performance would support the hypothesis of genetic purging or laboratory adaptation, whereas measurable phenotypic declines would suggest that deleterious alleles persist even within long-term colonies.

Our data revealed that although developmental durations differed statistically between the control and inbred groups across most ontogenetic stages, the absolute differences were marginal and rarely exceeded 1.2 days. While multi-generation inbreeding typically shortens insect lifespans [19], the developmental timelines observed here align with previously reported baseline data for BSF, where larval development spanned 17–28 days [20] and adult longevity ranged from 10 to 28 days under identical environmental conditions [21]. In a comparable study on a BSF line colonized for over 50 generations, the first generation of full-sib mating prolonged development by a non-significant ~ 2 days, whereas a substantial ~ 10-day delay emerged only in the second consecutive generation of inbreeding [18]. Furthermore, evidence from other insect systems suggests that minor shifts in developmental pacing can be driven by mass-rearing adaptations rather than inbreeding per se. For example, in the sweet potato weevil *Cylas formicarius elegantulus* (Coleoptera: Brentidae), a mass-reared strain exhibited consistently shorter developmental times than its wild counterpart, regardless of inbreeding or outbreeding status [22].

Although growth curves did not differ significantly between the groups, the inbred group exhibited higher substrate reduction and significantly lower ECI, alongside a non-significant increase in FCR. This discrepancy indicates that inbred individuals were less efficient at converting nutritional resources into biomass, driving up substrate consumption to satisfy elevated maintenance costs or alternative physicochemical and physiological processes. Across diverse insect species, such as *Gryllodes sigillatus* (Orthoptera: Gryllidae), inbreeding depression has been linked to increased energy expenditure and elevated metabolic rates [23]. Conversely, in other insects such as *Drosophila littoralis* (Diptera: Drosophilidae), inbreeding depression manifests as a suppressed metabolic rate accompanied by reduced offspring [24]. Irrespective of these inter-group variations, the baseline FCR, substrate reduction, and ECI values observed here remain highly consistent with previously documented ranges for BSF [19].

Differences in body weight between groups varied by developmental stage: the control larvae and adults were heavier than their inbred-group counterparts, whereas prepupal and pupal weights showed no significant difference (Fig. 3A), suggesting that inbreeding effects are specific to phases of resource accumulation and reproduction, as no noteworthy differences were identified during the prepupal and pupal stages. Both of these metamorphic stages are characterized by the cessation of feeding and extensive tissue reorganization. In our study, significant differences were confined to the larval and adult stages, where controls were heavier than inbred individuals. The manifestation of inbreeding depression in insects varies by species, trait, environment, and inbreeding coefficient, lacking a generalized effect on any specific life stage. For instance, inbred butterflies (*Heliconius erato phyllis*; Lepidoptera: Nymphalidae) exhibit reduced pupal weight and wing area [25], whereas inbred burying beetle larvae (*Nicrophorus orbicollis*; Coleoptera: Staphylinidae) remain lighter even when provided with abundant resources [26]. Interestingly, variations in adult physiological indicators were more strongly influenced by sex than by inbreeding status, with females consistently outweighing males in both groups. This finding aligns with previous documentation of pronounced sexual dimorphism in BSF, where females outweigh males by up to 20% [27, 28]. This baseline suggests that natural sexual dimorphism, rather than the degree of inbreeding, represents the primary source of body weight variance. However, the absence of an inbreeding effect on body weight does not imply that all fitness-related traits are unaffected. Evidence demonstrates that even when size, weight, or FCR remain unchanged by inbreeding, alternative traits can be highly sensitive [15, 29]. In the flour beetle *Tribolium castaneum* (Coleoptera: Tenebrionidae), for example, increased inbreeding is linked to reduced sperm size and smaller testes, thereby compromising reproductive success [30].

Fecundity traits in the evaluated BSF strain were largely unaffected by inbreeding, as no significant differences emerged in oviposition timing, duration, or overall fecundity between the control and inbred groups. This stability, contrasting with the observed physiological shifts, suggests that reproductive traits evaluated may be less susceptible to inbreeding depression, or that previous generations have already purged deleterious alleles affecting reproductive performance [8]. Although mean reproductive measures did not differ significantly between groups, considerable inter-individual clutch size variation was observed within both groups. Such phenotypic variability is well-documented across inbred insect lines, ranging from highly variable oviposition indicators, egg weights, or hatchability in the screwworm *Cochliomyia hominivorax* (Diptera: Calliphoridae) to a broader distribution of sperm length in *Anopheles gambiae* (Diptera: Culicidae) mosquitoes [31, 32]. Despite the descriptive differences in oviposition patterns and observed inter-individual variability in both groups, these results should be interpreted with caution given the absence of statistically significant differences.

In contrast to reproductive performance, a clearer effect emerged regarding BSF survival and longevity. Controls exhibited significantly longer lifespans than inbred individuals, suggesting that inbreeding depression can compromise adult survival even when previous developmental stages remain unaffected. Other dipterans, such as *Drosophila melanogaster* (Diptera: Drosophilidae) and *C. hominivorax*, exhibit substantial and progressive lifespan reductions across generations, particularly in females, with adult life expectancy dropping by up to 10% in *D. melanogaster* [29, 31, 33]. Interestingly, aligned with existing BSF literature [18], males outlived females in both groups in our single-generation comparison. This pattern also mirrors findings in *Callosobruchus maculatus* (Coleoptera: Chrysomelidae), where inbred males displayed longer lifespans than females at an inbreeding coefficient of *F* = 0.25 [21]. Additionally, the differences in LT_50_ values further illustrate that certain traits, specifically survival in female BSF, may be influenced by an interaction with energy allocation to reproduction, maintenance of the bodily functions and stress tolerance, potentially resulting in trade-offs among these physiological processes [23].

Although the phenotypic shifts observed after a full-sib mating were subtle across the evaluated temporal, physiological, and reproductive traits, several aspects warrant further investigation. Inbreeding depression often accumulates gradually over multiple successive generations, meaning short-term experiments conducted on recently domesticated strains may capture only the onset of long-term trajectories [22]. However, utilizing a strain already domesticated for more than 64 generations provides a highly valuable model to track these progressive changes, as the base population likely reached a high degree of genetic homozygosity. This advanced state of domestication might explain the limited differences observed, suggesting that potential deleterious alleles may have already been purged or fixed during the long-term laboratory maintenance. Importantly, because the precise genetic composition and baseline heterozygosity of our colony were not molecularly quantified, we cannot determine the exact inbreeding differential between the base population and the full-sib mating group. If the base population had already reached a high level of background inbreeding over these 64 generations, the actual genetic difference between the two experimental groups may have been small, which would further explain the subtle phenotypic shifts observed. While family-specific effects cannot be fully decoupled from the mating treatment due to the single-family origin of the inbred group, these findings provide important empirical evidence for the short-term tolerance of inbreeding within established BSF populations. Integrating molecular approaches, such as SNP genotyping and whole-genome sequencing, could elucidate the genetic mechanisms and consequences of inbreeding. Such analyses could quantify changes in heterozygosity, identify genomic regions under selection, and link phenotypic responses to underlying compensatory genetic mechanisms [8]. Importantly, this study utilized a laboratory-reared population maintained under stable conditions with minimal selection pressure. In contrast, industrial BSF strains are continuously selected for high-productivity traits and vary greatly in population size, genetic diversity, and baseline inbreeding levels. As reproductive output is influenced not only by genetics but also by husbandry practices, including cage density, environmental control, and mating conditions [34], this should be considered for further studies. Future research should compare these distinct rearing regimes to determine whether industrial selection pressures mitigate or amplify inbreeding depression. Research on inbreeding effects within highly domesticated or selectively bred BSF populations remains limited, as most inbreeding studies focus on wild or recently colonized groups [9, 10, 12, 13]. This represents a critical knowledge gap; prolonged captivity can trigger severe losses in genetic diversity and accelerate inbreeding, particularly when artificial selection drives adaptation to captive environments. Breeding for commercially desirable traits – such as accelerated larval growth, larger body size, or optimized feed conversion efficiency – frequently bottlenecks populations by selecting only a narrow subset of individuals to contribute to subsequent generations [12]. While simple phenotypic selection frequently accelerates this genetic erosion, advanced breeding designs and structured mating schemes can mitigate these effects by balancing selection response with the maintenance of genetic diversity. Given that BSF mass rearing has only expanded since the late 1980s [35] and genetic improvement programs have only recently emerged to meet commercial demands [36], many breeding lines have not yet been monitored over sufficient generations to fully evaluate the long-term impacts of domestication on genomic architecture and population fitness.

Lastly, while this study focused on well-established life-history parameters key to BSF production performance [16, 21, 23], alternative traits may serve as more sensitive indicators of inbreeding depression [15]. Evaluating parameters such as stress tolerance, immune competence, behavioral shifts, or reproductive fitness markers—including testes development, as well as sperm size and count—could unveil subtle inbreeding effects not captured by the phenotypic traits assessed here.

## Conclusions

This study evaluated the effects of inbreeding on the temporal, physiological, and reproductive traits of a laboratory BSF strain colonized for over 64 generations. Across the assessed parameters, full-sib mating did not trigger severe phenotypic declines compared to the control line. These findings indicate that acute, single-generation inbreeding does not substantially impair key fitness or production-related traits in this domesticated strain, demonstrating a high degree of robustness to genetic isolation under stable environmental conditions. This phenotypic resilience is highly relevant for both laboratory-maintained colonies and industrial rearing programs.

Crucially, these insights are valuable for managing sudden population bottlenecks, demonstrating that if a colony accidentally crashes and recovers from only a few surviving individuals, performance in mass-rearing systems may not be immediately compromised by the resulting inbreeding. However, because inbreeding effects can accumulate across subsequent generations or manifest in alternative physiological axes, investigating immune competence, stress resistance, and integrating genomic tools remains imperative. Collectively, this study addresses a critical knowledge gap in BSF domestication and provides a foundational population biology perspective for the optimization and long-term stabilization of industrial insect rearing systems.

## Methods

### Origin of the base population

BSF individuals for the experiments were randomly selected from a laboratory base population established in July 2018 from colony stock obtained from Bio.S Biogas (Grimma, Germany). Since its acquisition, this base population was continuously reared on commercially available chicken feed (CF; GoldDott Eierglück, DERBY Spezialfutter, Münster, Germany) and maintained in genetic isolation from other BSF populations for 64 generations. Each generation, the population size consisted of ~ 4000 adult individuals. Husbandry and environmental conditions remained constant for all generations leading up to this study. Colony maintenance was conducted in the greenhouse facility of the Fraunhofer Institute for Molecular Biology and Applied Ecology IME (Giessen, Germany), under controlled environmental conditions (27 ± 1 °C, 60 ± 5% relative humidity (RH), 12:12 h light: dark photoperiod).

### Establishment of the inbred line

Larvae hatched from 250 mg of eggs stemming from the base population were reared in polypropylene containers (19.5 × 16.5 × 9.5 cm; length (l)× width (w)× height(h)) and placed in a climate chamber at 27 ± 1 °C and 60 ± 5% RH in complete darkness. CF milled to a particle size of < 1.5 mm using a grain mill (Mockmill 200, Wolfgang Mock, Otzberg, Germany) was provided ad libitum as a larval diet. Once ≥ 50% of the individuals reached the sixth instar, prepupae were manually separated from the remaining frass and transferred into larger polypropylene containers (25 × 18 × 13 cm). For pupation and metamorphosis, these containers were placed inside mesh cages (30 × 30 × 30 cm; Bioform, Nuremberg, Germany) located in the greenhouse at 27 ± 1 °C, 60 ± 5% RH, and a 12:12 h light: dark photoperiod.

Within 24 h of adult emergence, virgin adults were collected using spring steel tweezers to prevent physical injury. Flies were sexed based on external dimorphisms of the antennae and abdominal genitalia [16]. To establish the full-sib family lines and obtain full-sib individuals for subsequent mating, ten temporally synchronized founder pairs were kept in the greenhouse. Each pair was isolated in an individual conical polypropylene container (8.4 × 8.4 × 11.4 cm) equipped with a circular 4.5 cm mesh insert for gas exchange. A 4 × 4 cm wooden board was included in each container to serve as an oviposition site, as described elsewhere [21]. Water was supplied daily by spraying 2 mL of tap water onto the inner walls of the containers, avoiding direct contact with the flies to prevent injury.

The presence of egg clutches on these founder pairs was monitored and documented daily. Of the ten founder pairs initially established, eight successfully oviposited, producing nine individual clutches, as one female oviposited twice. Egg clutches were collected separately and transferred into separate polypropylene containers (8.7 × 11.4 × 5.1 cm) to maintain family identity. These containers were subsequently placed in the climate chamber in complete darkness. After hatching, full-sib larvae were fed CF ad libitum and reared under the conditions described above until adult emergence. For subsequent full-sib mating, offspring originating from one founder pair was selected. From this family, 50 males and 50 females were selected following adult emergence to establish 50 separate full-sib mating pairs, each consisting of one male and one female sharing both parents. These pairs were maintained separately in conical polypropylene containers under the same conditions described for the founder pairs. Of these 50 full-sib mating pairs, one produced sufficient viable offspring to establish the experimental inbred group. A total of 500 larvae of this single mating pair were used for the experiment. After hatching, these larvae were provided with CF ad libitum until reaching a body length of 3–4 mm (third instar, L3; 5–10 days). Upon reaching this target size, larvae were counted and weighed using an AT261 DeltaRange analytical balance (Mettler, Giessen, Germany), which was subsequently used for all weight measurements throughout the study. The 500 inbred larvae were distributed into five experimental polypropylene containers (19.5 × 16.5 × 9.5 cm), each containing 100 individuals. Simultaneously, the dry matter (DM) content of the CF was determined in triplicate from 250 mg samples using an MA35 moisture analyzer (Sartorius, Göttingen, Germany). Each of the inbred and control containers was provisioned with 250 mg DM of CF per larva. This feed ration allowed the larvae to develop fully, ensuring that comparisons between replicates and groups were based solely on phenotypic growth potential without feed restrictions. The moisture content of the diet was adjusted to 67.5% using tap water. No additional feed or water was added throughout the experiment. The control group consisted of 3–4 mm larvae randomly obtained from the laboratory base population (described in Section “Origin of the base population”). Both experimental groups were maintained under identical feeding and environmental conditions.

### Comparison of life-history and morphological traits

All life-history and physiological traits described below were assessed using the experimental setup detailed in Section “Establishment of the inbred line” after establishment of the inbred line. Uniform environmental conditions and standardized measuring procedures were strictly maintained throughout the study to ensure direct comparability between the inbred and control groups.

#### Growth curves

For growth curve analysis, 25 larvae from each replicate container were randomly sampled daily using spring steel tweezers, and their pooled weight was recorded. Substrate residues were carefully removed from the larval cuticle prior to weighing. Following measurement, both the substrate residues and the larvae were immediately returned to their respective containers [16]. This procedure was repeated daily for each replicate until the pooled larval weight remained constant for three consecutive days. The third consecutive day of stable weight was defined as the endpoint of the growth curve.

#### Feed conversion ratio, efficiency of conversion of ingested feed, and substrate reduction

Once all larvae within a replicate reached the prepupal stage and the last individual was removed from the substrate, the remaining frass was collected, weighed, and its moisture content was determined in triplicate. According to Nayak et al. [17], the feed conversion ratio (FCR; Eq. 2), the efficiency of conversion of ingested feed (ECI; Eq. 1), and substrate reduction (SR; Eq. 3) were calculated using the DM of the feed and frass, paired with the fresh mass of the harvested larvae, which were kept alive for subsequent experiments.

1 $$\mathrm{FCR} = \frac{\text{Total feed provided (g)}}{\text{Total biomass harvested (g)} - \text{Initial biomass inoculated (g)}}$$

2 $$\mathrm{ECI} = \frac{(\text{Total biomass harvested (g)} - \text{Initial biomass inoculated (g)})}{\text{Total feed provided (g)} - \text{Frass residue (g)}} \times 100$$

3 $$SR = \frac{(\text{Total feed provided (g)} - \text{Frass residue (g)}) \times 100}{\text{Total feed provided (g)}}$$

#### Larval, prepupal, and pupal life-history indicators

Egg clutches, larvae, prepupae, and pupae were inspected daily at a fixed time to standardize the evaluation. Hatching was defined as the day on which ≥ 50% of an egg clutch hatched. Larval, prepupal, and intrapuparial development times were quantified via visual inspection as the total duration required for ≥ 50% of the surviving individuals to transition to the prepupal, pupal, and adult stages, respectively. Larvae were classified as prepupae when their bodies had developed complete dark-brown coloration, pupae were identified by complete immobility and a distinctly curved body shape [17]. The larva-to-prepupa and prepupa-to-pupa transition day was the first day on which at least 50% of the individuals alive in that container met this criterion.

#### Morphological traits

Morphological traits, including individual body weight and length, were monitored across the larval, prepupal, pupal, and adult stages. Surviving larvae within each replicate were separated from the frass at their peak body mass using spring steel tweezers, cleaned of surface impurities, counted, weighed and measured individually before returning to their feeding container. Peak body mass was defined as the third consecutive day where weight remained constant. The sum of the individual larval weights at this peak was defined as the total biomass at maximum larval mass. Body length was quantified using a Keyence VHX-5000 digital microscope (Keyence, Osaka, Japan), which was utilized for all optical measurements throughout the study. Larval length was measured in a relaxed position from the anterior tip of the head capsule to the posterior end of the eighth abdominal segment [16].

Following these peak measurements, individuals from each replicate of both groups were monitored daily for ontogenetic transitions. Newly transitioned prepupae (identified by a completely dark-brown coloration) and subsequent pupae were counted, recorded, and removed from their feeding container to a separate polypropylene container (19.5 × 16.5 × 9.5 cm) without food to await pupation and metamorphosis. Individual weight and body length were recorded on the day of transition. Prepupal length was measured in the same relaxed dorsal position as larvae, whereas pupal length was measured laterally from the tip of the head to the posterior end of the eighth abdominal segment. After pupation, individuals were maintained in 30 × 30 × 30 cm mesh cages located in the greenhouse at standard conditions. Within 24 h of emergence, virgin adults were collected using spring steel tweezers to prevent physical injury, weighed inside a 50 mL centrifugation tube (Greiner Bio-One, Frickenhausen, Germany), and sexed. Adult body length was determined post-mortem by measuring ventrally from the antennal pedicel to the subgenital plate [16].

#### Fecundity and reproductive indicators

Fecundity and reproductive life-history traits – including egg clutch production, oviposition timing, and adult longevity – were monitored throughout the adult stage. For each experimental group, 50 temporally synchronized mating pairs were established in individual conical containers (described in Section “Establishment of the inbred line”). Sex-specific longevity and the presence of egg clutches were documented daily. Adult longevity was measured as the number of days from emergence to death for each fly. Females were left undisturbed during the oviposition process. Individual egg clutches were collected on filter paper (MN 226, Macherey-Nagel, Düren, Germany) and weighed immediately.

The preoviposition period was defined as the duration from adult eclosion to the first oviposition event, whereas the oviposition period represented the timeframe from the first to the last oviposition event within a replicate [21]. The oviposition peak was defined as the day post-emergence on which the highest number of egg clutches was deposited. Adult survival describes the proportion of individuals remaining alive over time, in which the median lethal time (LT_50_) corresponded to the time from adult emergence to 50% cumulative mortality. Multiple oviposition events were recorded if a female deposited subsequent egg clutches at least 24 h after the primary oviposition. Fecundity was quantified as the proportion of females that laid eggs at least once, ensuring that multiple oviposition events by a single female were not counted repeatedly for this overall percentage. Number, timing, and mass of multiple oviposition events were recorded and evaluated separately. Egg clutch size (number of eggs per clutch) was estimated to have an average egg weight of 0.024 mg for flies reared on CF [18].

### Statistical analysis

Statistical analyses and data visualizations were performed using Excel 2016 (Microsoft, Redmond, WA, USA), OriginPro 2026 (OriginLab, Northampton, MA, USA), and R Statistical Software (v4.5.1; R Core Team 2025, Vienna, Austria). The Shapiro–Wilk test was applied to assess data normality, and Levene’s test was used to verify the homogeneity of variance. For mean comparisons between groups with normal distribution and homogeneous variances, a Student’s t-test (*t* statistics reported) was performed. If the sample data did not follow a normal distribution, a non-parametric Mann–Whitney U Test (*Z* statistics reported) was carried out. Two-way analysis of variance (ANOVA; *F* statistics reported) followed by Tukey’s post hoc test was utilized to evaluate oviposition events, sequential survival rates, as well as sex-specific adult lengths and weights. Adult lifetime data were assessed using the non-parametric Kaplan–Meier estimator and the S*(t)* survival functions were compared pairwise using log-rank tests (*χ²* statistics reported). Linear mixed-effects models (LMM) were employed to analyze differences in larval growth curve patterns. In the model, mean larval weight was defined as the response variable, with time and group (control versus inbred) treated as fixed effects. A comparison was made with a random intercept for each container, with a model that also allowed the effect of time to vary among containers. Model fit was compared using the Akaike Information Criterion (AIC). Degrees of freedom and *p* values for the fixed effects were estimated using the Satterwaite approximation (lmer function in lme4 package; [37]). An error level of *α* = 0.05 for statistical significance was set for all analyses Graphs were generated using the ggplot2 package in R Studio.

## Data Availability

The datasets used and/or analyzed during the current study are available from the corresponding author on reasonable request.
